# The use of linked routine data to optimise calculation of the Hospital Frailty Risk Score on the basis of previous hospital admissions: a retrospective observational cohort study

**DOI:** 10.1016/S2666-7568(21)00004-0

**Published:** 2021-03

**Authors:** Andrew Street, Laia Maynou, Thomas Gilbert, Tony Stone, Suzanne Mason, Simon Conroy

**Affiliations:** a Department of Health Policy, London School of Economics and Political Science, London, UK; b Center for Research in Health and Economics, Universitat Pompeu Fabra, Barcelona, Spain; c Hospices Civils de Lyon, Groupement Hospitalier Sud, Centre Hospitalier Lyon Sud, Lyon, France; d Connected Health Cities Urgent and Emergency Care Research group, School of Health and Related Research, University of Sheffield, Sheffield, UK; e Department of Health Sciences, University of Leicester, George Davies Centre, University Road, Leicester, UK

## Abstract

**Background:**

The Hospital Frailty Risk Score (HFRS) has been widely but inconsistently applied in published studies, particularly in how diagnostic information recorded in previous hospital admissions is used in its construction. We aimed to assess how many previous admissions should be considered when constructing the HFRS and the influence of frailty risk on long length of stay, in-hospital mortality, and 30-day readmission.

**Methods:**

This is a retrospective observational cohort study of patients aged 75 years or older who had at least one emergency admission to any of 49 hospital sites in the Yorkshire and Humber region of England, UK. We constructed multiple versions of the HFRS for each patient, each form incorporating diagnostic data from progressively more previous admissions in its construction within a 1-year or 2-year window. We assessed the ability of each form of the HFRS to predict long length of stay (>10 days), in-hospital death, and 30-day readmission.

**Findings:**

Between April 1, 2013, and March 31, 2017, 282 091 patients had 675 155 hospital admissions. Regression analyses assessing the different constructions of HFRS showed that the form constructed with diagnostic information recorded in the current and previous two admissions within the preceding 2 years performed best for predicting all three outcomes. Under this construction, 263 432 (39·0%) of 674 615 patient admissions were classified as having low frailty risk, for whom 33 333 (12·7%) had a long length of stay, 10 145 (3·9%) died in hospital, and 45 226 (17·2%) were readmitted within 30 days. By contrast with those patients with low frailty risk, for those with intermediate frailty risk, the probability was 2·5-times higher (95% CI 2·4 to 2·6) for long length of stay, 2·17-times higher (2·1 to 2·2) for in-hospital death, and 0·7% higher (0·5 to 1) for readmission. For patients with high frailty risk, the probability was 4·3-times higher (4·2 to 4·5) for long length of stay, 2·48-times higher (2·4 to 2·6) for in-hospital death, and −1% (−1·2 to −0·5) lower for readmission than those with low frailty risk. The intermediate and high frailty risk categories were more important predictors of long length of stay than any of the other rich set of control variables included in our analysis. These categories also proved to be important predictors of in-hospital mortality, with only the Charlson Comorbidity Index offering greater predictive power.

**Interpretation:**

We recommend constructing the HFRS with diagnostic information from the current admission and from the previous two admissions in the preceding 2 years. This HFRS form was a powerful predictor of long length of stay and in-hospital mortality, but less so of emergency readmissions.

**Funding:**

National Institute of Health Research.

## Introduction

Health systems worldwide are interested in identifying frailty in patients. Two widely used tools include the electronic Frailty Index (in primary care)^1^ and the Hospital Frailty Risk Score (HFRS; in secondary care).^2^ Gilbert and colleagues^2^ developed the HFRS focusing on individuals aged 75 years or older who are at greatest risk of harm and high resource use in hospital. This risk score takes values ranging from 0 (no frailty risk) to 173·2 and is calculated by combining a weighted set of 109 three-character diagnostic codes from the International Statistical Classification of Diseases and Related Health Problems, tenth revision (ICD-10), recorded during the current admission and any previous emergency admissions occurring in the preceding 2 years.

The HFRS has been widely adopted, but diagnostic information from past admissions is inconsistently used.^3^, ^4^, ^5^, ^6^, ^7^, ^8^, ^9^, ^10^, ^11^, ^12^, ^13^, ^14^, ^15^, ^16^, ^17^, ^18^, ^19^, ^20^, ^21^ At face value, using more information is always better, increasing the likelihood that the HFRS captures the true effect of frailty risk on patient outcomes. However, obtaining historical data might incur costs and, even if these costs were marginal, they might not be worth incurring if the extra data do not offer substantial additional information.

There are two forms of inconsistency in constructing the HFRS. First, variation occurs in the so-called look-back window (how far back to go in health records to search for data) in accounting for previous admissions. Some studies apply a 2-year window,^3^, ^4^, ^5^, ^6^ as recommended by Gilbert and colleagues.^2^ Some studies use information from the current admission alone,^7^, ^8^, ^9^, ^10^ whereas others look back over the preceding 3 months,^11^, ^12^ the preceding year,^13^, ^14^ and even up to the preceding 15 years.^15^, ^16^ In some studies, the look-back period is not specified.^17^, ^18^, ^19^ This variation in defining the look-back period entails a lack of external consistency in how the HFRS is constructed, compromising the comparison of findings across studies.

**Research in context**

**Evidence before this study**

We searched ResearchGate, ScienceDirect, Google scholar, and ClinicalTrials.gov using the search terms “Hospital Frailty Risk Score” (HFRS) and “HFRS” for studies in English published up to Oct 2, 2020, to identify studies that applied the HFRS to patients aged 75 or older to assess how past diagnostic data was used in its construction. Authors were contacted if these details were not reported in the article. We found that the HFRS has been widely but inconsistently applied, with studies differing in the use of diagnostic data from past emergency admissions when constructing the risk score.

**Added value of this study**

Our study offers two major contributions. First, we established what constitutes the minimum amount of diagnostic data from previous admissions required to construct the HFRS. More information offers better predictive accuracy, whether the estimated effects are larger or smaller than those based on less information, but historical data might be difficult to access. We determined the point at which there is no significant change in predictive ability, beyond which historical data are not worth accounting for. To determine this point, we constructed multiple forms of the HFRS and assessed their ability to predict long length of stay, in-hospital death, and 30-day emergency readmission. Second, we assessed the importance of the HFRS in predicting patient outcomes compared with a richer set of control variables than that used in the original HFRS study or in any other study that has since validated the HFRS, including patients' sociodemographic and clinical characteristics and their journey along the emergency and urgent care pathway before their admission to hospital.

**Implications of all the available evidence**

The HFRS should be constructed with diagnostic information recorded in the current and previous two admissions within the preceding 2 years, as diagnostic data from additional previous admissions did not add predictive value. The HFRS is a powerful predictor of long length of stay and in-hospital death, but not of 30-day readmission.

Second, studies will have internal inconsistency when constructing the HFRS if the look-back window had been defined by the period covered by the dataset but varied for individuals in the dataset.^20^, ^21^ Take a 2-year dataset, for instance: if a patient died at the start of these 2 years, they might only have a single record even though they might have been in hospital several times in the year or two before the start of the period covered by the data. For such an individual, their previous admissions would be unobserved and their frailty risk would be understated. By contrast, for a patient admitted towards the end of the data period, the frailty risk would be measured as intended by Gilbert and colleagues.^2^ In such applications, the HFRS will not be constructed consistently across patients in the same study.

In this study, we aimed to provide definitive guidance about how best to construct the HFRS both in terms of the number of previous admissions from which diagnostic data are extracted and the length of the look-back period so that the risk score can be used to predict accurately the probabilities of long length of stay (>10 days), dying in hospital, and 30-day readmission. To determine this minimum amount of previous admissions, we aimed to construct multiple forms of the HFRS and assessed their ability to predict long length of stay, in-hospital death, and 30-day emergency readmission. To assess the accuracy of HFRS predictions, we aimed to use a richer set of control variables than that used in the original HFRS study or in any other study that has since validated the HFRS. These control variables include patients' sociodemographic and clinical characteristics and their journey along the emergency and urgent care pathway before their admission to hospital.

## Methods

### Study setting and participants

We did a retrospective cohort study of individuals aged 75 years or older who had at least one emergency admission to any of the 49 sites of the 13 acute National Health Service (NHS) hospitals in the Yorkshire and Humber region of England (UK), with a population of 5·4 million people and a mixture of large and small urban, suburban, and rural settings. The Connected Health Cities Urgent and Emergency Care Research database (CUREd)^22^ collates routine data from NHS 111 service, the Yorkshire Ambulance Service computer-aided dispatch data, and the emergency department and inpatient patient administration systems. The full dataset runs from April 1, 2011, to March 31, 2017, but we ran the analyses from April 1, 2013, onwards so that the HFRS was always constructed consistently with use of a full 2-years' worth of historical data for all patients.

### Study design

In the first stage of the analyses, each patient was categorised as having low (HFRS <5), intermediate (5–15) or high (>15) frailty risk by constructing the HFRS with use of diagnostic information from the current admission alone (labelled HFRS_(a)_). We then ran regression analyses to assess the association between frailty risk and of long length of stay, in-hospital mortality, and 30-day emergency readmission, taking into account various control variables, including category variables for the intermediate-risk group (labelled HFRS^i^) and the high-risk group (labelled HFRS^h^), with patients in the low-risk group serving as the reference category.

In the second stage, we investigated how each individual's categorisation by frailty risk changed as progressively more past admissions occurring in the preceding year were considered in the construction of the HFRS. The second form of the HFRS, HFRS_(a + 1,1)_, captured diagnostic data from both the current and one previous admission (labelled a+1) that occurred within the preceding year (labelled 1). The third form, HFRS_(a + 2,1)_, included data from the current and previous two admissions occurring in the preceding year, and so on. One patient had 39 admissions in the year preceding their current admission, and this was captured by the final HFRS_(a + n,1)_ form.

**Figure 1:**
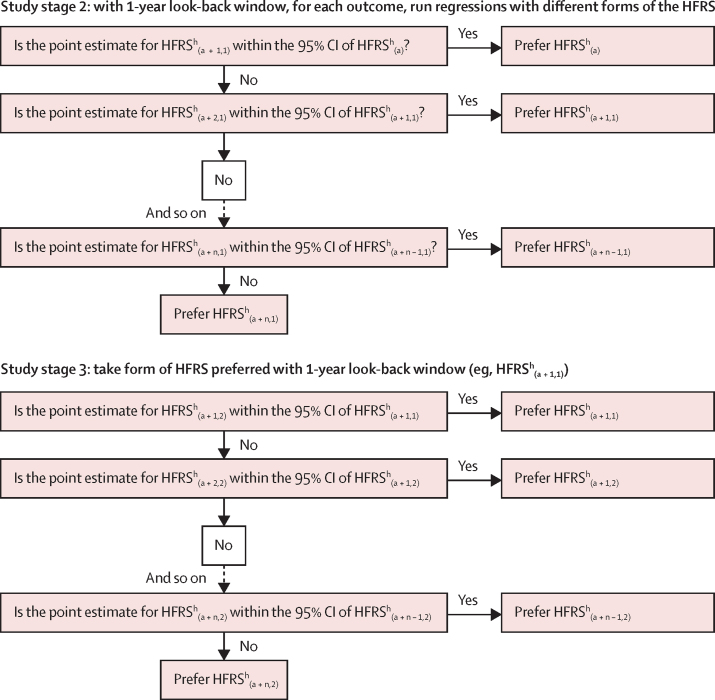
Decision rules to arrive at preferred form of HFRS (a)=current admission only. (a + n,1)=current admission and n previous admissions in the preceding year. HFRS=Hospital Frailty Risk Score.

To arrive at the preferred form of the HFRS, we assessed whether a significant (p<0·05) difference occurred in the point estimates associated with the high-risk frailty category under successive constructions of the HFRS, following the process set out in the top half of figure 1. If the point estimate from HFRS^h^_(a + 1,1)_ fell within the 95% CI of the point estimate for HFRS^h^_(a)_, then the HFRS_(a)_ construction was preferred, as the extra diagnostic data of HFRS^h^_(a + 1,1)_ did not increase the predictive ability compared with the use of fewer data. If not, we assessed whether the estimate for HFRS^h^_(a + 2,1)_ fell within the 95% CI of the estimate for HFRS^h^_(a + 1,1)_, and so on.

In the third stage, we repeated the preceding analyses after widening the look-back window in which previous admissions occurred to 2 years before the current admission, as set out in the lower half of figure 1. To determine whether to use a 1-year or 2-year look-back window, the 1-year version of the HFRS preferred in the second stage of analysis (eg, HFRS_(a + 1,1)_) was compared with its 2-year window version (eg, HFRS_(a + 1,2)_). If the point estimate from the 2-year version (eg, HFRS^h^_(a + 1,2)_) was within the 95% CI of the 1-year version, then the 1-year version was preferred. If not, the 2-year version was preferred because this additional diagnostic data yielded a significantly different estimate of the effect of frailty risk on each outcome. We also did a sensitivity analysis that assessed whether a 3-year window added predictive accuracy.

### Statistical analysis

The regression models took the general form

$$Y=f(HFRS^{i},HFRS^{h},X,P,T,Z)$$

where Y indicates one of three outcomes: long length of stay, in-hospital death, or 30-day emergency readmission. We used a Logit model for long length of stay, a Cox proportional hazard model for in-hospital death, and a sample selection bivariate probit model for 30-day readmission. As detailed in the appendix (p 4), the probit model for readmission recognises that in-hospital mortality is a competing risk for 30-day readmission and conditions the probability of readmission on whether the patient survived the previous hospitalisation.^23^ This involved first estimating a selection equation to explain the probability of survival before estimating the probability of readmission. This survival model accounts for the day of the admission, the argument being that the day of admission influences in-hospital mortality but has no bearing on the probability of readmission.^23^

If the regression coefficients for HFRS^i^ and HFRS^h^ were positive and significant, those patients with intermediate and high frailty risk had a higher likelihood of long length of stay (captured by odds ratios [ORs]), in-hospital death (captured by hazard ratios [HRs]), or emergency readmission (captured by average marginal effects) than patients assessed as having low frailty risk.

The regression analyses controlled for sociodemographic and clinical characteristics of patients, indicated by vector X, including age, sex, socioeconomic status, Charlson Comorbidity Index,^24^ number of emergency admissions in the preceding year, counts of the number of operation codes, whether or not they had ambulatory care-sensitive conditions (ACSC), the national tariff attached to the Healthcare Resource Group to which they were categorised, whether they were care home residents, and travel time between their residence and the hospital (appendix p 2).

Vector P includes variables capturing the patient's emergency and urgent care journey before admission: the number and length in minutes of the individual's emergency (NHS 111 and 999) calls, time of the ambulance on scene, time taken between calling the ambulance and arrival at the emergency department, the urgency with which the ambulance was dispatched, and whether the patient was admitted to hospital through the emergency department.

Variables indicating day, month, and year of admission were included in vector T. We included a fixed effect for each of the 49 hospital sites, represented as vector Z. For long length of stay and 30-day readmission, SEs were clustered at patient level to capture correlation across multiple admissions, perhaps to different hospital sites, by the same patient. Statistical analysis was done with Stata 15.

### Role of the funding source

The funders of the study had no role in study design, data collection, data analysis, data interpretation, or writing of the report.

## Results

The analysis sample comprised 282 091 patients who had 675 155 hospital admissions between April 1, 2013, and March 31, 2017. In-hospital death was recorded for all observations, discharge date was missing for 540 (0·1%) admissions for which neither long length of stay nor the HFRS could be calculated, and those patients censored at March 31, 2017, were assumed not to have been readmitted within 30 days. 177 255 (26·3%) of 674 615 observations had a long length of stay, 51 569 (7·6%) of 675 155 died in hospital, and 130 717 (19·4%) of 675 155 had an emergency readmission within 30 days of being discharged (table 1; summary statistics across all observations for each of the control variables are reported in appendix p 3). Regarding the HFRS_(a)_ frailty risk categorisation, 373 799 (55·4%) of 674 615 admissions were in the low category, 251 009 (37·2%) in the intermediate category, and 49 807 (7·4%) in the high category (table 1).

**Table 1:** Descriptive statistics of outcomes, 2013–17

		**Number of observations**	**Percentage**	**HFRS_(a)_**		
				Low	Intermediate	High
HFRS_(a)_ categorisation				373 799 (55·4%)	251 009 (37·2%)	49 807 (7·4%)
Outcomes						
	Length of stay (>10 days)	177 255/674 615	26·3% (44·1)	13·9% (34·6)	37·1% (48·3)	64·6% (47·8)
	In-hospital death	51 569/675 155	7·6% (26·6)	4·5% (20·8)	10·8% (31·1)	15·1% (35·7)
	Readmission	130 717/675 155	19·4% (39·5)	19·2% (39·4)	19·9% (39·9)	17·7% (38·2)

Data are n/N, n (%), or % (SD). In-hospital death was recorded for all observations. Discharge date was missing for 540 (0·1%) patients, for whom neither long length of stay nor the HFRS could be calculated, but they were included in the readmission analysis on the assumption that were not readmitted within 30 days. Of the 18 145 patients discharged after March 1, 2017, 1706 (9·4%) were readmitted before March 31, 2017, compared with a monthly average of 19·4% for the study period. The remaining censored observations were assumed not to have been readmitted within 30 days. HFRS=Hospital Frailty Risk Score. (a)=data from current admission alone.

The first stage of the study involved regression analysis of each of the three outcomes in turn, with frailty risk defined under the HFRS_(a)_ construction and including the control variables (full set of results in appendix p 6). Compared with patients with low frailty risk, those with intermediate risk were 2·66-times more likely to have a long length of stay and 1·96-times more likely to die in hospital, but 0·3% less likely to be readmitted within 30 days; those with high frailty risk were 5·47-times more likely to have a long length of stay, 2·24-times more likely to die in hospital, but 2·7% less likely to be readmitted within 30 days (table 2).

**Table 2:** Outcome coefficients for HFRS calculated with progressively more previous admissions

		**Length of stay (OR)**		**In-hospital deaths (HR)**		**30-day readmission (AME)**	
	HFRS intermediate	HFRS high	HFRS intermediate	HFRS high	HFRS	intermediate	HFRS high
Current admission alone (a)		2·66* (2·61 to 2·70)	5·47* (5·32 to 5·62)	1·96* (1·92 to 2·01)	2·24* (2·17 to 2·31)	−0·003* (−0·005 to −0·001)	−0·027* (−0·031 to −0·023)
1-year window							
	Current admission plus previous admission in preceding year (a + 1,1)	2·58* (2·53 to 2·63)	4·75* (4·64 to 4·87)	2·15* (2·10 to 2·21)	2·53* (2·45 to 2·61)	0·001 (−0·002 to 0·003)	−0·020* (−0·023 to −0·016)
	Current admission plus previous 2 admissions in preceding year (a + 2,1)	2·61* (2·56 to 2·66)	4·76* (4·64 to 4·88)	2·22* (2·16 to 2·28)	2·62* (2·54 to 2·71)	0·003* (0·001 to 0·006)	−0·017* (−0·020 to −0·013)
	Current admission plus previous 3 admissions in preceding year (a + 3,1)	2·64* (2·58 to 2·69)	4·80* (4·68 to 4·92)	2·24* (2·18 to 2·30)	2·63* (2·55 to 2·73)	0·005* (0·002 to 0·008)	−0·016* (−0·020 to −0·013)
	Current admission plus previous 4 admissions in preceding year (a + 4,1)	2·64* (2·59 to 2·70)	4·83* (4·71 to 4·96)	2·25* (2·18 to 2·31)	2·65* (2·57 to 2·74)	0·006* (0·004 to 0·009)	−0·013* (−0·017 to −0·010)
	Current admission plus previous n admissions in preceding year (a + n,1)	2·65* (2·60 to 2·70)	4·82* (4·70 to 4·94)	2·25* (2·19 to 2·31)	2·66* (2·57 to 2·75)	0·009* (0·006 to 0·011)	−0·004* (−0·007 to −0·000)
2-year window							
	Current admission plus previous admission in preceding 2 years (a + 1,2)	2·50* (2·45 to 2·55)	4·50* (4·39 to 4·61)	2·12* (2·06 to 2·17)	2·45* (2·37 to 2·53)	0·003* (0·001 to 0·006)	−0·015* (−0·018 to −0·012)
	Current admission plus previous 2 admissions in preceding 2 years (a + 2,2)	2·50* (2·45 to 2·55)	4·34* (4·24 to 4·45)	2·17* (2·11 to 2·23)	2·48* (2·40 to 2·56)	0·007* (0·004 to 0·010)	−0·009* (−0·012 to −0·005)
	Current admission plus previous 3 admissions in preceding 2 years (a + 3,2)	2·51* (2·46 to 2·56)	4·31* (4·21 to 4·42)	2·19* (2·12 to 2·25)	2·47* (2·39 to 2·56)	0·010* (0·007 to 0·012)	−0·006* (−0·009 to −0·003)
	Current admission plus previous 4 admissions in preceding 2 years (a + 4,2)	2·51* (2·46 to 2·57)	4·32* (4·21 to 4·42)	2·19* (2·13 to 2·26)	2·47* (2·39 to 2·55)	0·011* (0·009 to 0·014)	−0·003 (−0·006 to 0·001)
	Current admission plus previous n admissions in preceding 2 years (a + n,2)	2·52* (2·47 to 2·57)	4·26* (4·16 to 4·37)	2·20* (2·14 to 2·26)	2·47* (2·39 to 2·55)	0·014* (0·011 to 0·016)	0·010* (0·007 to 0·014)

Data are coefficients (95% CI), using low frailty risk as the reference category and after adjusting for all control variables. OR=odds ratio. HFRS=Hospital Frailty Risk Score. HR=hazard ratio. AME=average marginal effect.

* Coefficient is significant (p<0·05).

In the second stage, we repeated the regression analyses, progressively substituting the intermediate and high frailty risk categories constructed based on HFRS_(a)_ for constructions based on HFRS_(a + 1,1)_, HFRS_(a + 2,1)_, HFRS_(a + 3,1)_, HFRS_(a + 4,1)_, and HFRS_(a + n,1)_, using increasingly more previous admissions occurring in the preceding year. The proportion of patients in the low frailty risk category decreased and the proportion of those in the high-risk category increased as data from progressively more historical admissions was used to construct the HFRS (table 3).

**Table 3:** Proportion of patients in each frailty risk category under different forms of the HFRS

		**Low frailty risk**	**Intermediate frailty risk**	**High frailty risk**
Current admission alone (a)		373 799 (55·4%)	251 009 (37·2%)	49 807 (7·4%)
HFRS 1-year window				
	Current admission plus previous admission in preceding year (a + 1,1)	301 882 (44·7%)	270 652 (40·1%)	102 081 (15·1%)
	Current admission plus previous 2 admissions in preceding year (a + 2,1)	283 891 (42·1%)	262 129 (38·9%)	128 595 (19·1%)
	Current admission plus previous 3 admissions in preceding year (a + 3,1)	278 364 (41·3%)	256 593 (38·0%)	139 658 (20·7%)
	Current admission plus previous 4 admissions in preceding year (a + 4,1)	276 330 (41·0%)	254 017 (37·7%)	144 268 (21·4%)
	Current admission plus previous n admissions in preceding year (a + n,1)	274 419 (40·7%)	251 491 (37·3%)	148 705 (22·0%)
HFRS 2-year window				
	Current admission plus previous admission in preceding 2 years (a + 1,2)	288 411 (42·8%)	275 278 (40·8%)	110 926 (16·4%)
	Current admission plus previous 2 admissions in preceding 2 years (a + 2,2)	263 432 (39·0%)	263 944 (39·1%)	147 239 (21·8%)
	Current admission plus previous 3 admissions in preceding 2 years (a + 3,2)	254 826 (37·8%)	254 875 (37·8%)	164 914 (24·4%)
	Current admission plus previous 4 admissions in preceding 2 years (a + 4,2)	251 347 (37·3%)	249 942 (37·0%)	173 326 (25·7%)
	Current admission plus previous n admissions in preceding 2 years (a + n,2)	247 640 (36·7%)	243 487 (36·1%)	183 488 (27·2%)

Percentages are proportions of a total of 674 615 observations. HFRS=Hospital Frailty Risk Score.

**Figure 2:**
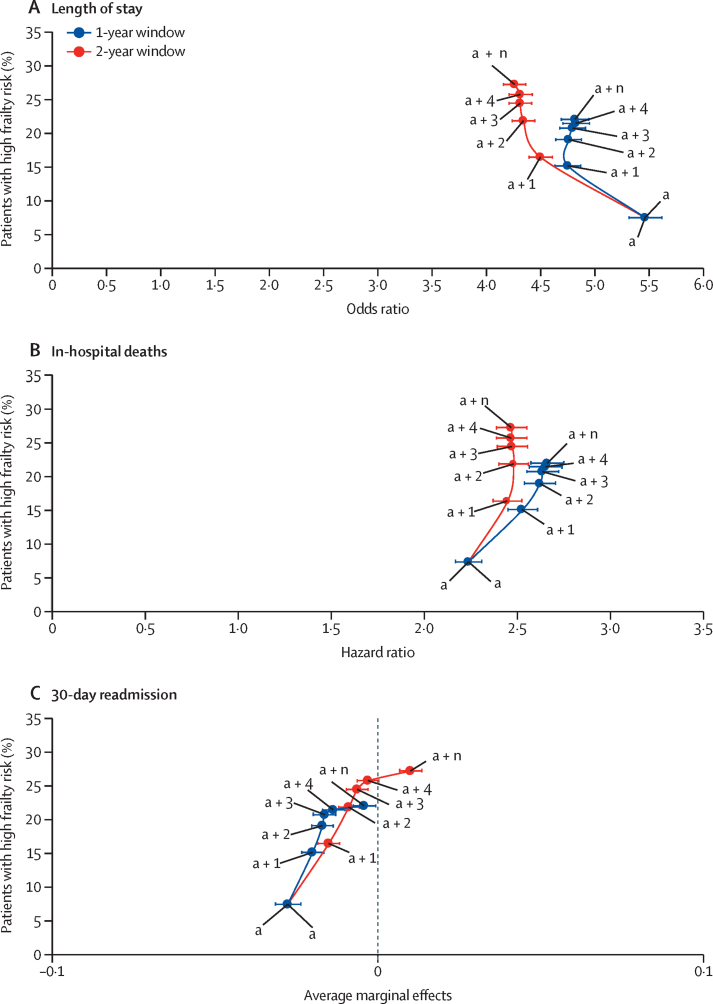
HFRS^h^ estimates for length of stay (>10 days; A), in-hospital deaths (B), and 30-day readmissions (C) within 1 year and 2 years (a)=current admission. (a + n)=current admission and n previous admissions. h=high-risk group. HFRS=Hospital Frailty Risk Score.

We plotted the proportion of patients in the high frailty risk category against the OR (95% CI) for long length of stay under each version of the HFRS (figure 2A). When adding one previous admission within a 1-year window to the current admission for the construction of the HFRS (HFRS_(a + 1,1)_), the proportion of patients with high frailty risk increased and the adjusted OR decreased significantly (OR of HFRS^h^_(a + 1,1)_ was outside the 95% CI of HFRS^h^_(a)_; table 2). By contrast, changes in the point estimates for each subsequent increase in the number of previous admissions (HFRS^h^_(a + 2,1)_, HFRS^h^_(a + 3,1)_, HFRS^h^_(a + 4,1)_, and HFRS^h^_(a + n,1)_) were not significant (table 2); therefore, no significant change was observed in predictive power associated with the use of diagnostic data from any of these past admissions to construct the HFRS. This indicates that, if restricted to a single year's worth of data, data from the current and previous admission (HFRS_(a + 1,1)_) are sufficient to predict long length of stay. Applying the same strategy, we found that the preferred construction was HFRS_(a + 2,1)_ for in-hospital death and HFRS_(a + 1,1)_ for 30-day readmissions (figure 2B, C).

In the third stage, we tested whether using a 2-year window offered greater accuracy. We observed that, compared with a 1-year window, a 2-year window resulted in larger proportions of patients categorised as having high frailty risk under each form of the HFRS (table 3).

We plotted the proportion of patients in the high frailty risk category against the OR (95% CI) for long length of stay under each version of the 2-year window HFRS (figure 2A). Using a 2-year window, the predictive power of high frailty risk on long length of stay was lower than that predicted when using a 1-year window (table 2). Within a 1-year window, the HFRS^h^_(a + 1,1)_ was preferred for predicting long length of stay. The point estimate for the 2-year form, HFRS^h^_(a + 1,2)_, was outside the 95% CI for HFRS^h^_(a + 1,1)_, indicating that predictive accuracy improved with the use of 2-years' worth of data (table 2; figure 2A). Assessing the subsequent versions of HFRS, we then established that the point estimate for HFRS^h^_(a + 2,2)_ was outside the 95% CI for HFRS^h^_(a + 1,2)_, whereas the point estimates for HFRS^h^_(a + 3,2)_, HFRS^h^_(a + 4,2)_, and HFRS^h^_(a + n,2)_ all were within the 95% CI for HFRS^h^_(a + 2,2)_. These findings imply that, to predict long length of stay accurately, it is best to construct HFRS_(a + 2,2)_, the version that uses diagnostic data from the current and previous two admissions that occurred in the preceding 2 years.

For in-hospital deaths and 30-day readmissions, our analyses reached the same conclusion as for long length of stay, with the point estimate for HFRS^h^_(a + 2,2)_ lying outside the 95% CI for HFRS^h^_(a + 2,1)_ (figure 2B, C). These findings mean that the HFRS_(a + 2,2)_ construction was the optimal form of the HFRS for all three outcomes. The HFRS_(a + 2,2)_ form was also preferred when the look-back window was widened to 3 years, with the predictions for the 2-year and 3-year windows almost exactly the same for all three outcomes (appendix p 12). This finding implies that no additional significant information was gained by applying a 3-year compared with a 2-year window. By extension, this ruled out the need to look even further back in time.

Under this HFRS_(a + 2,2)_ form, of the 674 615 observations, 263 432 (39·0%) were classified as low frailty risk, 263 944 (39·1%) as intermediate frailty risk, and 147 239 (21·8%) as high frailty risk. For the 263 432 observations with low frailty risk, 33 333 (12·7%) resulted in a long length of stay, 10 145 (3·9%) in in-hospital death, and 45 226 (17·2%) in readmission within 30 days. Compared with observations with low frailty risk, the probability for observations with intermediate frailty risk was 2·5-times higher for long length of stay (95% CI 2·4 to 2·6), 2·17-times higher for in-hospital death (2·1 to 2·2), and 0·7% higher for readmission (0·5% to 1). For observations with high frailty risk, the probability was 4·3-times higher (4·2 to 4·5) for long length of stay, 2·48-times higher (2·4 to 2·6) for in-hospital death, and −1% (−1·2 to −0·5) lower for readmission, compared with observations with low frailty risk.

**Figure 3:**
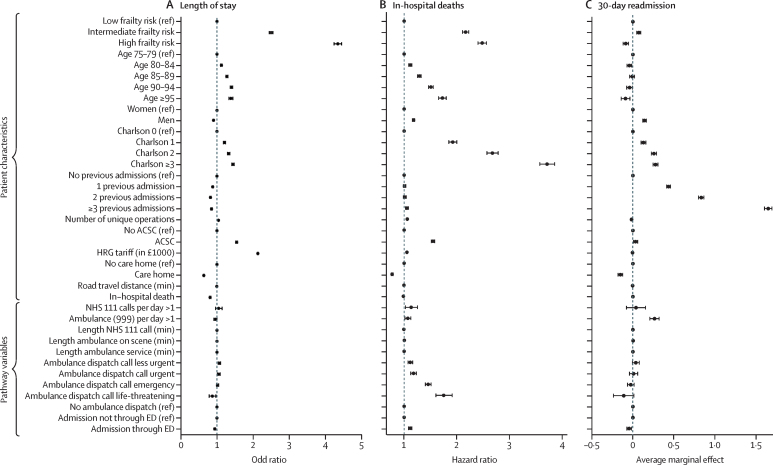
Regression results for length of stay (>10 days; A), in-hospital deaths (B), and 30-day readmissions (C) for HFRS_(a + 2,2)_ (a + 2,2)=current admission plus 2 previous admissions in the preceding 2 years. ACSC=ambulatory care-sensitive conditions. ED=emergency department. HFRS=Hospital Frailty Risk Score. HRG=Healthcare Resource Group. NHS=National Health Service.

Full regression results for the three outcomes when using the HFRS_(a + 2,2)_ construction are reported in the appendix (p 7) and summarised as forest plots (figure 3). The intermediate and high frailty risk categories were more important predictors of long length of stay than any of the other patient or pathway characteristics considered, none of which influenced long length of stay by more than 2·2-times their reference categories (figure 3A). The importance of the HFRS as a predictor held when examining the probability of length of stay in excess of 7 days and 21 days (appendix pp 8–9). For predicting in-hospital death, the Charlson Comorbidity Index was the most important (particularly for scores of 3 up to the maximum of 24 [HR 3·71]), followed by the frailty risk categories (figure 3B). The probability of dying in hospital also increased with age (HR 1·73 for patients older than 95 years) and for patients who were admitted with an ACSC (HR 1·55), and it increased with increasing urgency of the patient's condition as designated by the call handler (HR 1·75 for patients with a life-threatening condition; figure 3B).

The probability of 30-day readmission was higher for more previous admissions to hospital (17% higher if three or more in the preceding year) and for higher Charlson scores (2·7% higher for scores of three or higher; figure 3C). Patients living in care homes were 1·5% less likely to be readmitted. Compared with patients with low frailty risk, those with intermediate frailty risk were 1% more likely to be readmitted and those with high frailty risk were 1% less likely to be readmitted (figure 3C).

## Discussion

The HFRS has been widely applied, but studies have inconsistently used previous admissions in its construction. In our study, we considered various forms of the HFRS to determine the minimum amount of diagnostic data to be used. Our assessment was based on whether there are significant changes in the ability of the high frailty risk marker to predict long length of stay, in-hospital death, and 30-day readmission in our cohort of patients. We judged that predictive ability was not improved if the HFRS^h^ point estimate from a form of the HFRS constructed with more diagnostic data was within the 95% CI of the HFRS^h^ from the preceding version. We found that the preferred form of the HFRS to predict all three outcomes used diagnostic data from the current and previous two admissions in the preceding 2-years.

Being categorised as having intermediate or high frailty risk was a more important predictor of long length of stay than any of the other rich set of control variables included in our analysis. These categories also proved to be important predictors of in-hospital mortality, with only the Charlson Comorbidity Index offering greater predictive power. The observation that both the HFRS and Charlson categories were highly significant predictors when jointly considered shows that they capture different risk factors associated with in-hospital death. By contrast, frailty risk was not a strong predictor of 30-day readmission. The inability of the HFRS to predict readmission might be because of the care that patients who are frail receive: if post-discharge care packages are put in place to support these individuals when they return to their usual place of residence, then their risk of readmission might be reduced.

Our findings that the HFRS is a strong predictor of long length of stay and in-hospital death are in line with other studies that have applied the HFRS to older people.^2^, ^4^ In agreement with some studies,^3^, ^4^ but not with others,^2^, ^10^ we found that the HFRS is not a strong predictor of readmission. Our study offers key advantages that make our predictions of frailty risk on outcomes more precise than those reported by other studies. The CUREd dataset covers 7 years, but data from the first 2 years were used solely to ensure that the HFRS was constructed consistently for all individuals. We benefited from a large sample size, thereby generating more precise estimates than those for small samples. Additionally, by linking individual data across emergency and urgent care settings, we controlled for a rich set of variables that might be correlated with frailty risk. Not accounting for these influences might over-estimate the impact of frailty risk on the outcomes.

Our study has limitations. The control variables can be improved, for instance by accounting for ethnicity—known to be poorly coded in English hospital data^25^—and by use of better measures of socioeconomic status. The positive association between the HFRS and longer length of stay might be upwardly biased because patients with longer stays tend to have more extensive documentation of frailty-related diagnoses,^26^ although the use of three-character rather than four-character ICD-10 codes offers some protection against this bias. The HFRS might not be feasible in health-care systems that do not routinely collect ICD-10 codes electronically. Although not a national study, our data are from a large, representative region of the UK.

Although no frailty tool discriminates well enough to direct clinical care at the individual patient level, tools can be used to identify cohorts at greater risk of poor outcomes.^27^ If embedded into hospital electronic health records, the HFRS could be used to target frailty interventions, avoiding the burden of implementing manual scores such as the Clinical Frailty Scale and improving the standardisation of frailty assessment. The combination of the electronic Frailty Index for primary care and HFRS for secondary care risk stratification would advance frailty-friendly care, as called for in various policy documents.^28^ The electronic Frailty Index and HFRS identify different at-risk cohorts, and thus their combination offers opportunity to identify the whole population at risk. Further research could examine how well the HFRS performs in other health-care systems, refine the HFRS through the incorporation of real-time data, and develop frailty-attuned clinical decision support systems.^29^

## Data sharing

Retrospective, deidentified data were obtained from the CUREd Research database (CUREdRQ0004) hosted by the CURE Group (University of Sheffield, Sheffield, UK). Data were provided under a data sharing agreement which prohibits onward sharing. Other researchers can make their own requests for CUREd data online.
