# Supplementary material for: The use of linked routine data to optimise calculation of the Hospital Frailty Risk Score on the basis of previous hospital admissions: a retrospective observational cohort study
Source: Lancet Healthy Longev. 2021 Mar;2(3):e154–62. doi: 10.1016/S2666-7568(21)00004-0 (PMC7934406; doi:10.1016/S2666-7568(21)00004-0)
Supplement: Supplementary appendix [file mmc1.pdf]

THE LANCET
Healthy Longevity

Supplementary appendix

This appendix formed part of the original submission and has been peer reviewed. We post it as supplied by the authors.

Supplement to: Street A, Maynou L, Gilbert T, et al. The use of linked routine data to optimise calculation of the Hospital Frailty Risk Score on the basis of previous hospital admissions: a retrospective observational cohort study. *Lancet Healthy Longev* 2021; published online Feb 23. https://doi.org/10.1016/S2666-7568(21)00004-0.

Appendix.Using linked routine data to optimise calculation of the Hospital Frailty Risk Score based on prior hospital admissions: an observational cohort study

Andrew Street[1], Laia Maynou[1,2], Thomas Gilbert[3], Tony Stone[4], Suzanne Mason[4] and Simon Conroy[5]

[1]Department of Health Policy, London School of Economics and Political Science, Houghton Street, WC2A 2AE London, UK

[2]Center for Research in Health and Economics (CRES), Universitat Pompeu Fabra, Ramon Trias Fargas 25-27 08005 Barcelona, Spain

[3]Hospices Civils de Lyon, Groupement Hospitalier Sud, Centre Hospitalier Lyon Sud, 165 chemin du Grand Revoyet 69495, Pierre-Bénite Cedex

[4]CURE group, School of Health and Related Research, University of Sheffield, S1 4DA, UK

[5]Department of Health Sciences, University of Leicester, George Davies Centre, University Road, Leicester, LE1 7RH, UK

A Appendix

A.1 Descriptive statistics

In addition to the patient´s frailty risk score, the analyses controlled for a set of socio-demographic and clinical characteristics, indicated by vector X in the regression model. These variables include the patients' age, categorized into 5-year age bands; sex; and the socioeconomic conditions of where they lived using the deciles of Index of Multiple Deprivation (IMD) [1], with IMD=1 indicating the worst-off communities; the Charlson comorbidity index [2] which uses age and ICD-10 indicators of comorbidity to estimate mortality risk and taking values from 0 to 17 but categorised for the analysis into four groups (0,1,2 and 3+); number of emergency admissions in the past year; counts of the number of operation codes; whether or not they had Ambulatory Care Sensitive Conditions (ACSC) [3]; the national tariff attached to the Healthcare Resource Group (HRG) to which they were categorized; whether patients were care home residents; for the travel time by road between the patient´s residence and the hospital [4].

Vector P in the regression model includes variables capturing the patient's emergency and urgent care journey prior to admission, namely: the number and length in minutes of the individual´s emergency (NHS111 and 999) calls; time of the ambulance on scene (arrival to departure); time taken between calling the ambulance and arrival at the emergency department; the urgency with which the ambulance was dispatched, assigned by the NHS Pathways triage system based on answers from the caller to scripted questions asked by the call-handler; and whether the patient was admitted to hospital through the ED.

Vector T in the regression model includes variables accounting for the day of hospital admission and whether this was on a public holiday [5]; and month and year variables capturing seasonal effects and annual trends. These variables are not reported in the tables. In the analysis of LoS, we accounted for whether the patient died in hospital. In the analyses of in-hospital death and 30-day readmission, we accounted for the patient´s LoS during the current admission.

Table A1 reports the descriptive statistics. The reference patient had low frailty risk, age 75-79, female, IMD 1, Charlson=0, no previous admission, no ACSC, was not a care home resident, made no NHS111 or 999 calls, did not require an ambulance and was not admitted through the ED.

Table A1: Descriptive statistics: covariates, 2013-2017

Variables	N	Mean/Proportion	Std. Dev.	Min	Max
**Patient characteristics**					
Age	674,540	83.748	5.796	75	120
Age 75-79	674,540	0.280	0.449	0	1
Age 80-84	674,540	0.293	0.455	0	1
Age 85-89	674,540	0.248	0.432	0	1
Age 90-94	674,540	0.138	0.345	0	1
Age 95+	674,540	0.039	0.195	0	1
Sex (=1 male)	675,149	0.430	0.495	0	1
IMD_ 1 decile	673,003	0.170	0.375	0	1
IMD_ 2 decile	673,003	0.101	0.302	0	1
IMD_ 3 decile	673,003	0.108	0.311	0	1
IMD_ 4 decile	673,003	0.085	0.279	0	1
IMD_ 5 decile	673,003	0.091	0.287	0	1
IMD_ 6 decile	673,003	0.100	0.300	0	1
IMD_ 7 decile	673,003	0.103	0.304	0	1
IMD_ 8 decile	673,003	0.088	0.283	0	1
IMD_ 9 decile	673,003	0.086	0.280	0	1
IMD_ 10 decile	673,003	0.069	0.253	0	1
Charlson comorbidity Index	675,155	2.021	1.876	0	17
Charlson comorbidity Index =0	675,155	0.209	0.407	0	1
Charlson comorbidity Index =1	675,155	0.274	0.446	0	1
Charlson comorbidity Index =2	675,155	0.202	0.402	0	1
Charlson comorbidity Index =3+	675,155	0.314	0.464	0	1
Number of previous admissions (one-year window)	674,615	1.173	1.766	0	39
Number of previous admissions (one-year window) =0	674,615	0.475	0.499	0	1
Number of previous admissions (one-year window) =1	674,615	0.247	0.431	0	1
Number of previous admissions (one-year window) =2	674,615	0.127	0.333	0	1
Number of previous admissions (one-year window) =3+	674,615	0.151	0.358	0	1
Num unique operations (OPCS)	674,615	1.284	2.399	0	31
ACSC (=1 yes)	675,155	0.194	0.396	0	1
HRG tariff (in 1,000 pounds)	674,615	2.760	3.130	0.162	148.781
Care Home (=1 yes)	673,280	0.163	0.369	0	1
Road travel distance (min) (LSOA to hospital)	669,434	13.842	9.314	0	63.65
**Pathway variables**					
Calls to NHS111 per day (>1)[1]	62,830	0.070	0.256	0	1
Ambulance (999) per day (>1)[1]	402,586	0.046	0.209	0	1
Length NHS111 call (min)[1]	62,830	14.23	13.030	1.133	73.06
Length ambulance on scene (min)[1]	391,507	39.817	18.895	0	103.87
Length ambulance service (min)[1]	402,585	86.424	44.898	0.183	238.76
Ambulance dispatched - Less-urgent	402,586	0.498	0.500	0	1
Ambulance dispatched - Urgent	402,586	0.181	0.385	0	1
Ambulance dispatched - Emergency	402,586	0.311	0.463	0	1
Ambulance dispatched - Life-threatening	402,586	0.010	0.098	0	1
Admission through ED (=1 yes)	675,155	0.777	0.416	0	1

Notes: [1]In the regressions, we assign 0 minutes to the rest of the observations.

A.2 Sample selection bivariate probit model

The sample selection bivariate probit model for 30-day readmission recognises that in-hospital mortality is a competing risk for 30-day readmission and conditions the probability of readmission on whether the patient survived the previous hospitalisation [6]. This involved first estimating a selection equation to explain the probability of survival before estimating the probability of readmission. The survival model accounts for the day of the week of the admission, the argument being that this influences in-hospital mortality but has no bearing on the probability of readmission [6].

Accounting for day of the week accords with the literature showing that there is a “weekend effect” associated with hospital survival, perhaps because of differences in staffing levels and mix across the week [7; 8]. Hence the survival equation recognises that the probability of in-hospital mortality varies by day of admission day-to-day.

In contrast, the day of the week that the patient was originally admitted has no bearing on the probability that they will be readmitted to hospital 30 days later. The argument by Laudicella et al is that the day of the week of the original admission “can be assumed to be uncorrelated with the risk of a readmission, which depends on post-operative care that can be provided more flexibly over a long period of time once survival has been assured” [6]. For our data, the proportionate shares across days of the week are almost identical for all patients and for those who are re-admitted, as shown in Figure A1. This confirms that the day of the original admission does not influence the probability of readmission.

Figure A1: Day of week of original admission

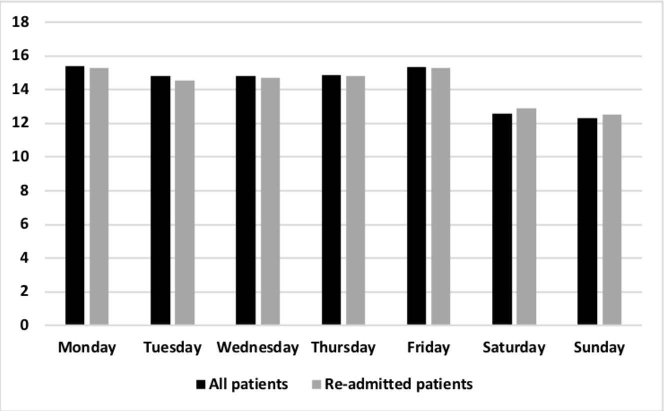

Some studies, including our original analysis [9], resolve the competing risk problem simply by excluding patients who died in hospital when estimating 30-day readmission. We assessed this as a sensitivity analysis, reported in column (4) of Table A4 and as a forest plots in Figure A2. Obviously, this regression includes fewer patients, but the impact on the size of the regression coefficients

is minimal and the conclusion that the HFRS is of little value in predicting readmission remains unchanged.

A.3 Regression results and sensitivity analysis

The starting point for our analyses was the construction of HFRS using diagnostic information recorded in the current admission only, ie HFRS(a). Applying this construction, Table A2 reports results for intermediate and high frailty risk markers denoted respectively as $HFRS^i_{(a)}$ and $HFRS^h_{(a)}$ and for the variables included in vectors X and P (the time variables in vector T are included in the regressions but results are not reported).

Our analyses indicated that the preferred form of the HFRS should be based on diagnostic data recorded in the current admissions and previous two admissions if these occurred in the past two years, ie HFRS(a+2,2). Table A3 reports results for $HFRS^i_{(a+2,2)}$ and $HFRS^h_{(a+2,2)}$ and the vectors of X and P variables.

Table A3 reports sensitivity analyses, all applying the HFRS(a+2,2) form of the HFRS. In our main analysis we defined long LoS using a 10-day cut-off for consistency with the original Gilbert et al paper [9], where it was chosen because of its relation to NHS England policy at the time. Current policy in England focuses on 7-day and 21-day cut-offs, as set out in Getting It Right First Time (GIRFT) standards for geriatric medicine [10]. Regression results from models based on the 7-day cut-off point are reported in column (1) and for the 21-day cut-off in column (3), with the 10-day results reproduced in column (2) for comparison. Forest plots for the 7-day and 21-day cut-offs are shown in Figure A2. In both Forest plots the importance of the intermediate and high risk frailty markers, $HFRS^i$ and $HFRS^h$, at explaining LoS is clearly evident, especially so for those with LoS in excess of 21 days.

Table A2: Regression results: HFRS(a) construction

Dep Var:	(1) LoS (>10 days)	(2) In-hospital death	(3) 30-day readmission
	**Odds ratio**	**Hazard ratio**	**Bivariate prob. AME**
**Intermediate Frailty Risk**	2.658***	1.962***	-0.003***
	(0.0231)	(0.0224)	(0.001)
**High Frailty Risk**	5.469***	2.238***	-0.027***
	(0.0772)	(0.0369)	(0.002)
**Age 80-84**	1.143***	1.136***	-0.004***
	(0.0127)	(0.0151)	(0.001)
**Age 85-89**	1.306***	1.319***	-0.0003
	(0.0148)	(0.0179)	(0.002)
**Age 90-94**	1.444***	1.543***	-0.003*
	(0.0188)	(0.0236)	(0.002)
**Age 95+**	1.442***	1.780***	-0.008***
	(0.0296)	(0.0382)	(0.003)
**Sex (=1 male)**	0.902***	1.174***	0.014***
	(0.00739)	(0.0109)	(0.001)
**IMD 2**	0.997	0.945***	-0.008***
	(0.0157)	(0.0169)	(0.002)
**IMD 3**	1.008	0.930***	-0.003
	(0.0157)	(0.0165)	(0.002)
**IMD 4**	1.001	0.935***	-0.006***
	(0.0169)	(0.0176)	(0.002)
**IMD 5**	0.987	0.925***	-0.008***
	(0.0163)	(0.0174)	(0.002)
**IMD 6**	1.020	0.882***	-0.012***
	(0.0165)	(0.0162)	(0.002)
**IMD 7**	1.011	0.914***	-0.013***
	(0.0161)	(0.0166)	(0.002)
**IMD 8**	0.988	0.907***	-0.015***
	(0.0166)	(0.0175)	(0.002)
**IMD 9**	0.971*	0.898***	-0.014***
	(0.0169)	(0.0176)	(0.002)
**IMD 10**	0.973	0.831***	-0.018***
	(0.0185)	(0.0182)	(0.002)
**Charlson =1**	1.181***	1.921***	0.013***
	(0.0145)	(0.0387)	(0.001)
**Charlson =2**	1.291***	2.685***	0.026***
	(0.0167)	(0.0547)	(0.002)
**Charlson 3+**	1.370***	3.684***	0.029***
	(0.0167)	(0.0718)	(0.002)
**Previous adm (one year) =1**	1.120***	1.146***	0.043***
	(0.0104)	(0.0132)	(0.001)
**Previous adm (one year) =2**	1.155***	1.202***	0.081***
	(0.0134)	(0.0166)	(0.002)
**Previous adm (one year) 3+**	1.184***	1.238***	0.163***
	(0.0138)	(0.0166)	(0.002)
**Num unique oper**	1.044***	1.059***	-0.002***
	(0.002)	(0.002)	(0.0002)
**ACSC (=1 Yes)**	1.587***	1.560***	0.003**
	(0.015)	(0.016)	(0.001)
**HRG tariff (in 1,000 pounds)**	2.092***	1.053***	-0.001*
	(0.007)	(0.002)	(0.0003)
**Care home (=1 Yes)**	0.643***	0.781***	-0.015***
	(0.007)	(0.009)	(0.001)
**Road travel distance (min)**	0.995***	0.996***	-0.0004***
	(0.001)	(0.001)	(5.91e-05)
**In-hospital death**	0.776***		
	(0.011)		
**LoS (days)**		0.985***	-4.65e-05
		(0.001)	(6.19e-05)
**NHS111 calls per day (>1)**	1.044	1.128**	0.004
	(0.047)	(0.058)	(0.006)
**Amb (999) per day (>1)**	0.954**	1.062**	0.026***
	(0.0221)	(0.028)	(0.003)
**Length NHS111 call (min)**	1.000	0.995***	-7.07e-05
	(0.001)	(0.001)	(8.74e-05)
**Length amb on scene (min)**	1.002***	1.003***	0.0002***
	(0.0003)	(0.0003)	(3.49e-05)
**Length amb service (min)**	1.001***	0.999***	2.15e-05
	(0.0001)	(0.0002)	(1.64e-05)
**Amb disp - less-urgent**	1.076***	1.127***	0.004*
	(0.017)	(0.023)	(0.002)
**Amb disp - urgent**	1.063***	1.184***	0.001
	(0.021)	(0.027)	(0.003)
**Amb disp - emergency**	1.050***	1.481***	-0.003
	(0.017)	(0.028)	(0.002)
**Amb disp - life-threatening**	0.899*	1.768***	-0.012*
	(0.050)	(0.080)	(0.006)
**Admission through ED**	0.913***	1.103***	-0.004***
	(0.010)	(0.015)	(0.001)
**N**	668,834	277,625	668,850
**Site fixed-effects**	Yes	Yes	Yes
**Time fixed-effects (day,month,year)**	Yes	Yes	Yes
**S.E. cluster**	Patient	robust	Patient
**Years**	2013-2017	2013-2017	2013-2017

Notes: Significance levels: *** p<0.01, ** p<0.05, * p<0.1. Reference categories: Low Frailty Risk, Age 75-79, Female, IMD 1, Charlson=0, Previous adm (one year)=0, No ACSC, No care home, NHS111 calls per <1, Amb (999) per day <1, No ambulance dispatched and Admission not through ED.

Table A3: Regression results: HFRS (a+2,2) construction

Dep Var:	(1)<br>LoS (>10 days)	(2)<br>In-hospital death	(3)<br>30-day readmission
	**Odds ratio**	**Hazard ratio**	**Bivariate prob. AME**
**Intermediate Frailty Risk**	2.496***	2.166***	0.007***
	(0.026)	(0.031)	(0.001)
**High Frailty Risk**	4.343***	2.480***	-0.009***
	(0.054)	(0.041)	(0.002)
**Age 80-84**	1.122***	1.116***	-0.004***
	(0.012)	(0.015)	(0.001)
**Age 85-89**	1.274***	1.287***	-0.001
	(0.014)	(0.018)	(0.002)
**Age 90-94**	1.402***	1.507***	-0.004**
	(0.018)	(0.023)	(0.002)
**Age 95+**	1.384***	1.725***	-0.009***
	(0.028)	(0.037)	(0.003)
**Sex (=1 male)**	0.905***	1.177***	0.014***
	(0.007)	(0.011)	(0.001)
**IMD 2**	1.008	0.950***	-0.008***
	(0.016)	(0.017)	(0.002)
**IMD 3**	1.013	0.935***	-0.003
	(0.016)	(0.017)	(0.002)
**IMD 4**	1.007	0.937***	-0.006***
	(0.017)	(0.018)	(0.002)
**IMD 5**	0.997	0.932***	-0.008***
	(0.016)	(0.018)	(0.002)
**IMD 6**	1.032**	0.889***	-0.012***
	(0.016)	(0.016)	(0.002)
**IMD 7**	1.019	0.922***	-0.013***
	(0.016)	(0.017)	(0.002)
**IMD 8**	0.995	0.908***	-0.015***
	(0.017)	(0.018)	(0.002)
**IMD 9**	0.982	0.902***	-0.014***
	(0.017)	(0.018)	(0.002)
**IMD 10**	0.989	0.838***	-0.018***
	(0.019)	(0.018)	(0.002)
**Charlson =1**	1.209***	1.922***	0.013***
	(0.015)	(0.039)	(0.001)
**Charlson =2**	1.326***	2.675***	0.026***
	(0.017)	(0.055)	(0.002)
**Charlson =3+**	1.441***	3.713***	0.027***
	(0.017)	(0.072)	(0.002)
**Previous adm (one year) =1**	0.884***	1.007	0.043***
	(0.009)	(0.012)	(0.001)
**Previous adm (one year) =2**	0.819***	1.013	0.083***
	(0.010)	(0.014)	(0.002)
**Previous adm (one year) =3+**	0.850***	1.052***	0.165***
	(0.010)	(0.015)	(0.002)
**Num unique oper**	1.042***	1.059***	-0.002***
	(0.002)	(0.002)	(0.0002)
**ACSC (=1 Yes)**	1.544***	1.547***	0.003**
	(0.014)	(0.016)	(0.001)
**HRG tariff (in 1,000 pounds)**	2.133***	1.053***	-0.001**
	(0.007)	(0.002)	(0.0003)
**Care home (=1 Yes)**	0.637***	0.772***	-0.015***
	(0.007)	(0.009)	(0.001)
**Road travel distance (min)**	0.995***	0.996***	-0.0004***
	(0.001)	(0.0005)	(5.91e-05)
**In-hospital death**	0.813***		
	(0.012)		
**LOS (days)**		0.986***	-0.0001**
		(0.001)	(6.10e-05)
**NHS111 calls per day (>1)**	1.048	1.134**	0.004
	(0.046)	(0.058)	(0.006)
**Amb (999) per day (>1)**	0.958*	1.069**	0.026***
	(0.022)	(0.028)	(0.003)
**Length NHS111 call (min)**	1.000	0.994***	-6.64e-05
	(0.001)	(0.001)	(8.74e-05)
**Length amb on scene (min)**	1.002***	1.003***	0.0002***
	(0.0003)	(0.0003)	(3.49e-05)
**Length amb service (min)**	1.001***	0.999***	2.06e-05
	(0.0001)	(0.0002)	(1.64e-05)
**Amb disp - less-urgent**	1.066***	1.117***	0.004*
	(0.017)	(0.022)	(0.002)
**Amb disp - urgent**	1.052***	1.177***	0.001
	(0.021)	(0.027)	(0.003)
**Amb disp - emergency**	1.016	1.455***	-0.003
	(0.0160)	(0.028)	(0.002)
**Amb disp - life-threatening**	0.873**	1.751***	-0.011*
	(0.048)	(0.079)	(0.006)
**Admission through ED**	0.940***	1.114***	-0.005***
	(0.010)	(0.016)	(0.001)
**N**	668,834	277,625	668,850
**Site fixed-effects**	Yes	Yes	Yes
**Time fixed-effects (day,month,year)**	Yes	Yes	Yes
**S.E. cluster**	Patient	robust	Patient
**Years**	2013-2017	2013-2017	2013-2017

Notes: Significance levels: *** p<0.01, ** p<0.05, * p<0.1. Reference categories: Low Frailty Risk, Age 75-79, Female, IMD 1, Charlson=0, Previous adm (one year)=0, No ACSC, No care home, NHS111 calls per <1, Amb (999) per day <1, No ambulance dispatched and Admission not through ED.

Table A4: Sensitivity analysis. Regression results: LoS and 30-day readmission. HFRS (a+2,2) construction

Dep Var:	(1) LoS (>7 days)	(2) LoS (>10 days)	(3) LoS (>21 days)	(4) 30-day readmission
	Odds ratio	Odds ratio	Odds ratio	Logit AME
Intermediate Frailty Risk	2.189***	2.496***	3.244***	0.0151***
	(0.0201)	(0.0258)	(0.0547)	(0.00137)
High Frailty Risk	3.385***	4.343***	7.721***	0.00336*
	(0.0387)	(0.0537)	(0.144)	(0.00182)
Age 80-84	1.096***	1.122***	1.099***	-0.00285*
	(0.0111)	(0.0124)	(0.0175)	(0.00154)
Age 85-89	1.231***	1.274***	1.204***	0.00303*
	(0.0129)	(0.0144)	(0.0195)	(0.00165)
Age 90-94	1.340***	1.402***	1.317***	0.00449**
	(0.0163)	(0.0181)	(0.0242)	(0.00195)
Age 95+	1.370***	1.384***	1.267***	0.00562*
	(0.0260)	(0.0279)	(0.0356)	(0.00307)
Sex (=1 male)	0.895***	0.905***	0.978*	0.0180***
	(0.00677)	(0.00731)	(0.0112)	(0.00120)
IMD 2	1.002	1.008	1.019	-0.00873***
	(0.0146)	(0.0157)	(0.0223)	(0.00240)
IMD 3	1.005	1.013	1.025	-0.00374
	(0.0143)	(0.0156)	(0.0223)	(0.00234)
IMD 4	0.994	1.007	1.007	-0.00643***
	(0.0154)	(0.0167)	(0.0238)	(0.00249)
IMD 5	0.982	0.997	0.978	-0.00925***
	(0.0150)	(0.0162)	(0.0225)	(0.00253)
IMD 6	1.005	1.032**	0.976	-0.0134***
	(0.0149)	(0.0164)	(0.0220)	(0.00235)
IMD 7	0.992	1.019	0.978	-0.0142***
	(0.0145)	(0.0160)	(0.0218)	(0.00241)
IMD 8	0.968**	0.995	0.966	-0.0164***
	(0.0151)	(0.0165)	(0.0227)	(0.00245)
IMD 9	0.965**	0.982	0.984	-0.0152***
	(0.0153)	(0.0168)	(0.0235)	(0.00244)
IMD 10	0.965**	0.989	0.976	-0.0197***
	(0.0170)	(0.0185)	(0.0253)	(0.00263)
Charlson =1	1.181***	1.209***	1.286***	0.0165***
	(0.0130)	(0.0148)	(0.0234)	(0.00154)
Charlson =2	1.258***	1.326***	1.461***	0.0337***
	(0.0147)	(0.0170)	(0.0277)	(0.00169)
Charlson =3+	1.338***	1.441***	1.565***	0.0473***
	(0.0148)	(0.0174)	(0.0278)	(0.00163)
Previous adm (one year) =1	0.906***	0.884***	0.814***	0.0458***
	(0.00801)	(0.00845)	(0.0113)	(0.00127)
Previous adm (one year) =2	0.856***	0.819***	0.749***	0.0875***
	(0.00957)	(0.00978)	(0.0128)	(0.00179)
Previous adm (one year) =3+	0.871***	0.850***	0.778***	0.172***
	(0.00983)	(0.0101)	(0.0129)	(0.00247)
Num unique oper	1.047***	1.042***	1.011***	-0.000206
	(0.00188)	(0.00190)	(0.00237)	(0.000253)
ACSC (=1 Yes)	1.617***	1.544***	1.438***	0.0127***
	(0.0139)	(0.0142)	(0.0195)	(0.00143)
HRG tariff (in 1,000 pounds)	2.566***	2.133***	1.787***	0.000108
	(0.0108)	(0.00726)	(0.00462)	(0.000303)
Care home (=1 Yes)	0.697***	0.637***	0.574***	-0.0143***
	(0.00728)	(0.00720)	(0.00926)	(0.00157)
Road travel distance (min)	0.995***	0.995***	0.993***	-0.000409***
	(0.000440)	(0.000472)	(0.000670)	(6.45e-05)
In-hospital death	0.759***	0.813***	0.865***	
	(0.0104)	(0.0116)	(0.0167)	
LoS (days)				-0.000126*
				(6.81e-05)
NHS111 calls per day (>1)	1.108**	1.048	1.111*	0.00737
	(0.0456)	(0.0460)	(0.0676)	(0.00632)
Amb (999) per day (>1)	0.939***	0.958*	0.986	0.0287***
	(0.0203)	(0.0221)	(0.0309)	(0.00306)
Length NHS111 call (min)	0.999**	1.000	1.001	-0.000195**
	(0.000614)	(0.000661)	(0.000937)	(9.39e-05)
Length amb on scene (min)	1.002***	1.002***	1.000	0.000247***
	(0.000243)	(0.000259)	(0.000365)	(3.73e-05)
Length amb service (min)	1.001***	1.001***	1.001***	1.16e-05
	(0.000113)	(0.000120)	(0.000171)	(1.75e-05)
Amb disp - less-urgent	1.072***	1.066***	1.089***	0.00424*
	(0.0161)	(0.0170)	(0.0251)	(0.00225)
Amb disp - urgent	1.034*	1.052***	1.173***	0.00428
	(0.0188)	(0.0206)	(0.0334)	(0.00273)
Amb disp - emergency	1.009	1.016	1.177***	0.00475**
	(0.0147)	(0.0159)	(0.0265)	(0.00220)
Amb disp - life-threatening	0.888**	0.873**	1.001	0.00742
	(0.0446)	(0.0475)	(0.0809)	(0.00732)
Admission through ED	0.960***	0.940***	0.895***	-0.00317**
	(0.00962)	(0.0101)	(0.0142)	(0.00151)
N	668,836	668,834	668,834	617,587
Site fixed-effects	Yes	Yes	Yes	Yes
S.E. cluster	Patient	Patient	Patient	Patient
Years	2013-2017	2013-2017	2013-2017	2013-2017

Notes: Significance levels: *** p<0.01, ** p<0.05, * p<0.1. Reference categories: Low Frailty Risk, Age 75-79, Female, IMD 1, Charlson=0, Previous adm (one year)=0, No ACSC, No care home, NHS111 calls per <1, Amb (999) per day <1, No ambulance dispatched and Admission not through ED.

Figure A2: Sensitivity analysis. Regression results for LoS (>7 days), LoS (>7 days) and 30-day readmissions (logit) - HFRS(a+2,2)

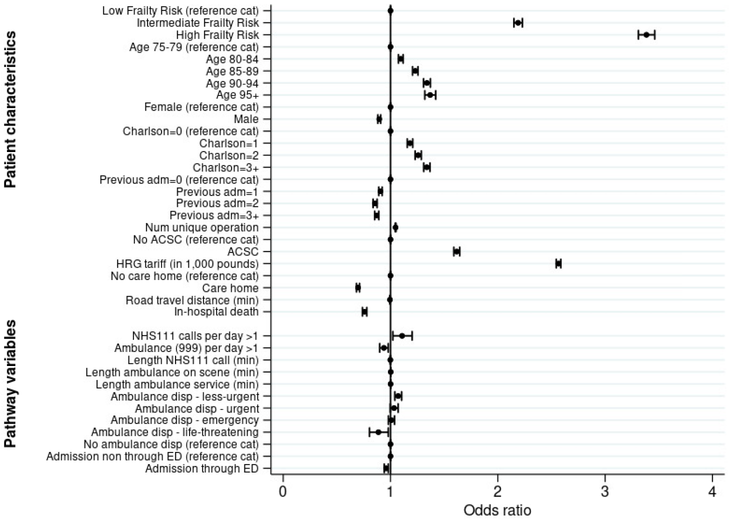

(I) - LoS (>7 days)

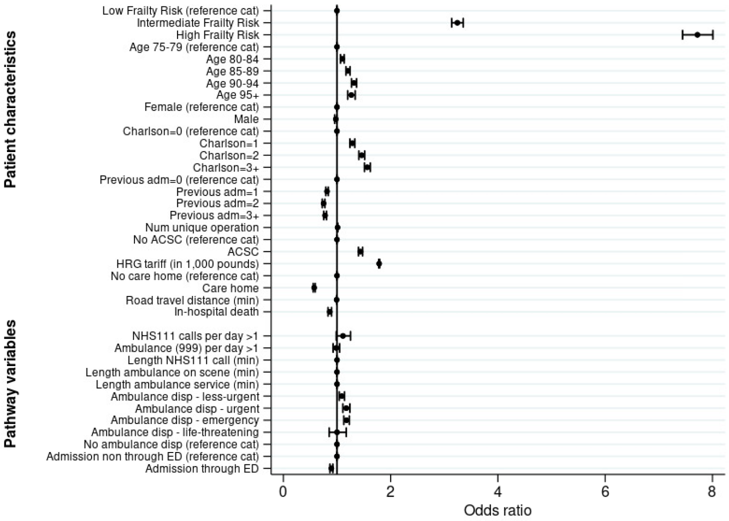

(II) - LoS (>21 days)

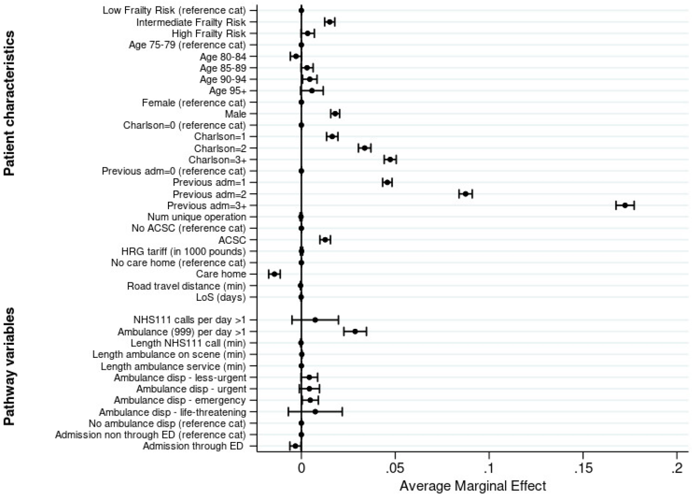

(III) - 30-day readmissions

A.4 HFRS 3-year window

Table A5 reports the proportions in each frailty risk category under each construction of the HFRS when applying one-year, two-year and three-year look-back windows while Table A6 reports the regression coefficients for $HFRS^i$ and $HFRS^h$ for each HFRS form.

These coefficients and 95% CIs for $HFRS^h$ are summarised in Figure A3 by the black line for the one-year window, the grey line for the two-year window and the dotted line for the three-yeear window. This demonstrates that the HFRS(a+2,2) form is preferred if the look-back window is widened to three years, the lines for the two- and three-year windows tracking each other almost exactly for all three outcomes. This implies that no additional significant information was gained by applying a three-year compared to two-year window. By extension, this rules out the need to look even further back in time when constructing the HFRS.

Table A5: Proportions in each frailty risk category under different forms of the HFRS

HFRS	Low	Intermediate	High
**Panel A: One-year window**			
**a**	55.4%	37.2%	7.4%
**a+1,1**	44.8%	40.1%	15.1%
**a+2,1**	42.1%	38.9%	19%
**a+3,1**	42.3%	38%	20.7%
**a+4,1**	41%	37.6%	21.4%
**...**	...	...	...
**a+n,1**	40.7%	37.3%	22%
**Panel B: Two-year window**			
**a**	55.4%	37.2%	7.4%
**a+1,2**	42.8%	40.8%	16.4%
**a+2,2**	39.1%	39.1%	21.8%
**a+3,2**	37.8%	37.8%	24.4%
**a+4,2**	37.3%	37%	25.7%
**...**	...	...	...
**a+n,2**	36.7%	36.1%	27.2%
**Panel C: Three-year window**			
**a**	55.4%	37.2%	7.4%
**a+1,3**	42%	41.1%	16.9%
**a+2,3**	37.8%	39.3%	22.9%
**a+3,3**	36.3%	37.7%	26%
**a+4,3**	35.6%	36.8%	27.6%
**...**	...	...	...
**a+n,3**	34.9%	35.4%	29.7%

Notes: a denotes current admission and n is the total number of previous admissions within the last year, the last two years or the last three years.

Table A6: Outcome coefficients for HFRS calculated with progressively more previous admissions

Panel A: One-year window	HFRS	a	a+1,1	a+2,1	a+3,1	a+4,1	...	a+n,1
LoS (OR)	Intermediate	**2.66**	**2.58**	**2.61**	**2.64**	**2.64**	...	**2.65**
		(2.61-2.70)	(2.53-2.63)	(2.56-2.66)	(2.58-2.69)	(2.59-2.70)	...	(2.60-2.70)
	High	**5.47**	**4.75**	**4.76**	**4.80**	**4.83**	...	**4.82**
		(5.32-5.62)	(4.64-4.87)	(4.64-4.88)	(4.68-4.92)	(4.71-4.96)	...	(4.70-4.94)
In-hospital deaths (HR)	Intermediate	**1.96**	**2.15**	**2.22**	**2.24**	**2.25**	...	**2.25**
		(1.92-2.01)	(2.10-2.21)	(2.16-2.28)	(2.18-2.30)	(2.18-2.31)	...	(2.19-2.31)
	High	**2.24**	**2.53**	**2.62**	**2.63**	**2.65**	...	**2.66**
		(2.17-2.31)	(2.45-2.61)	(2.54-2.71)	(2.55-2.73)	(2.57-2.74)	...	(2.57-2.75)
30-day readmission (AME)	Intermediate	**-0.003**	0.001	**0.003**	**0.005**	**0.006**	...	**0.009**
		(-0.005- -0.001)	(-0.002-0.003)	(0.001-0.006)	(0.002-0.008)	(0.004-0.009)	...	(0.006-0.011)
	High	**-0.027**	**-0.020**	**-0.017**	**-0.016**	**-0.013**	...	**-0.004**
		(-0.031- -0.023)	(-0.023- -0.016)	(-0.020- -0.013)	(-0.020- -0.013)	(-0.017- -0.010)	...	(-0.007- -0.000)
**Panel B: Two-year window**	**HFRS**	**a**	**a+1,2**	**a+2,2**	**a+3,2**	**a+4,2**	**...**	**a+n,2**
LoS (OR)	Intermediate	**2.66**	**2.50**	**2.50**	**2.51**	**2.51**	...	**2.52**
		(2.61-2.70)	(2.45-2.55)	(2.45-2.55)	(2.46-2.56)	(2.46-2.57)	...	(2.47-2.57)
	High	**5.47**	**4.50**	**4.34**	**4.31**	**4.32**	...	**4.26**
		(5.32-5.62)	(4.39-4.61)	(4.24-4.45)	(4.21-4.42)	(4.21-4.42)	...	(4.16-4.37)
In-hospital deaths (HR)	Intermediate	**1.96**	**2.12**	**2.17**	**2.19**	**2.19**	...	**2.20**
		(1.92-2.01)	(2.06-2.17)	(2.11-2.23)	(2.12-2.25)	(2.13-2.26)	...	(2.14-2.26)
	High	**2.24**	**2.45**	**2.48**	**2.47**	**2.47**	...	**2.47**
		(2.17-2.31)	(2.37-2.53)	(2.40-2.56)	(2.39-2.56)	(2.39-2.55)	...	(2.39-2.55)
30-day readmission (AME)	Intermediate	**-0.003**	**0.003**	**0.007**	**0.010**	**0.011**	...	**0.014**
		(-0.005- -0.001)	(0.001-0.006)	(0.004-0.010)	(0.007-0.012)	(0.009-0.014)	...	(0.011-0.016)
	High	**-0.027**	**-0.015**	**-0.009**	**-0.006**	-0.003	...	**0.010**
		(-0.031- -0.023)	(-0.018- -0.012)	(-0.012- -0.005)	(-0.009- -0.003)	(-0.006-0.001)	...	(0.007-0.014)
**Panel C: Three-year window**	**HFRS**	**a**	**a+1,2**	**a+2,2**	**a+3,2**	**a+4,2**	**...**	**a+n,2**
LoS (OR)	Intermediate	**2.66**	**2.48**	**2.47**	**2.47**	**2.47**	...	**2.48**
		(2.61-2.70)	(2.44-2.53)	(2.42-2.52)	(2.42-2.52)	(2.42-2.52)	...	(2.43-2.54)
	High	**5.47**	**4.47**	**4.27**	**4.22**	**4.20**	...	**4.12**
		(5.32-5.62)	(4.36-4.57)	(4.17-4.38)	(4.11-4.32)	(4.09-4.30)	...	(4.02-4.23)
In-hospital deaths (HR)	Intermediate	**1.96**	**2.11**	**2.14**	**2.15**	**2.16**	...	**2.16**
		(1.92-2.01)	(2.05-2.16)	(2.08-2.20)	(2.09-2.21)	(2.10-2.22)	...	(2.10-2.23)
	High	**2.24**	**2.43**	**2.44**	**2.41**	**2.40**	...	**2.38**
		(2.17-2.31)	(2.36-2.51)	(2.36-2.52)	(2.33-2.48)	(2.32-2.48)	...	(2.30-2.46)
30-day readmission (AME)	Intermediate	**-0.003**	**0.004**	**0.008**	**0.011**	**0.013**	...	**0.015**
		(-0.005- -0.001)	(0.002-0.006)	(0.006-0.011)	(0.009-0.014)	(0.010-0.015)	...	(0.013-0.018)
	High	**-0.027**	**-0.014**	**-0.006**	-0.003	0.001	...	**0.015**
		(-0.031- -0.023)	(-0.017- -0.010)	(-0.009- -0.002)	(-0.006-0.001)	(-0.003-0.004)	...	(0.012-0.019)

Notes: The table reports coefficients and 95% Confidence Intervals in brackets, using low frailty risk as the reference category and after adjusting for all control variables. Coefficients in bold are significant ($p<0.05$). OR=Odds Ratio, HR=Hazard Ratio, AME=Average Marginal Effect.

Figure A3: $HFRS^h$ estimates for LoS (>10 days), in-hospital deaths and 30-day readmissions (within 1 year, 2 years and 3 years)

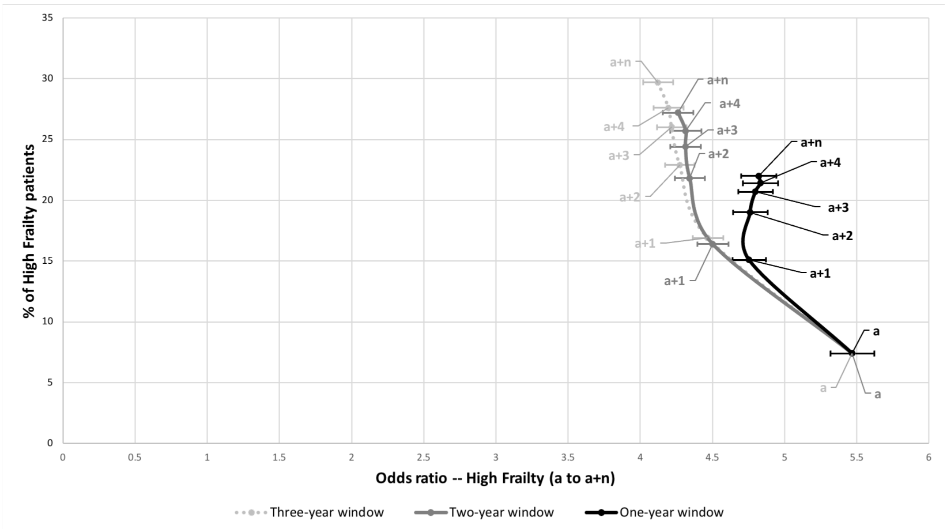

(I) - LoS (>10 days)

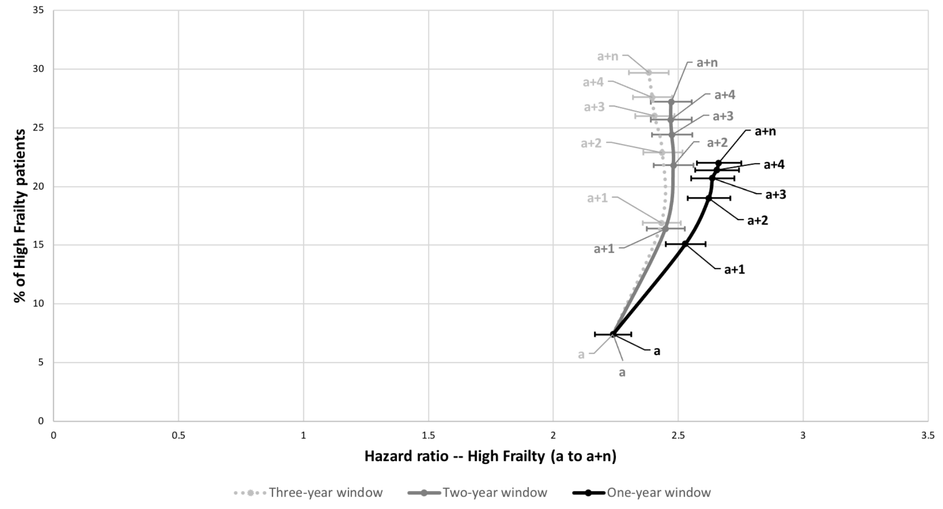

(II) - In-Hospital deaths

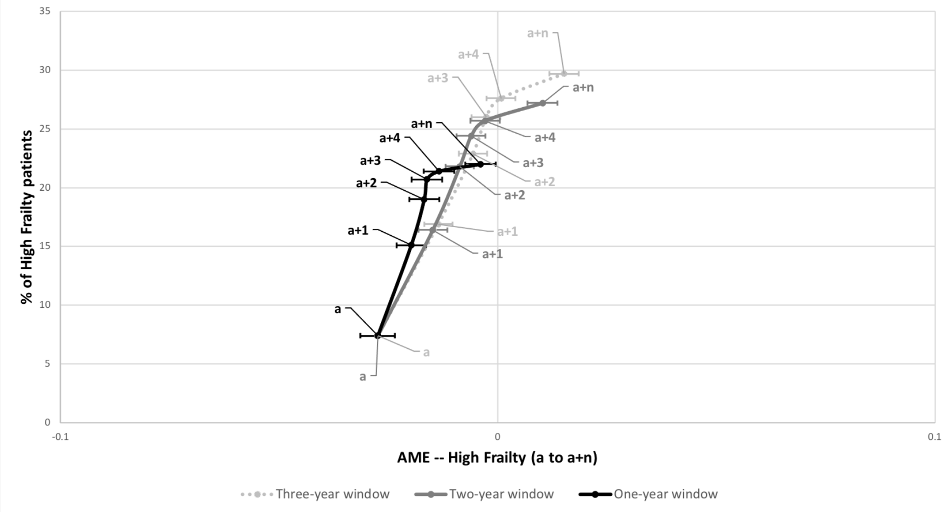

(III) - 30-day readmissions

References

[1] Ministry of Housing, Communities and Local Government. The English Indices of Deprivation 2015; 2015. [accessed: 12.06.2020]. https://www.gov.uk/government/statistics/english-indices-of-deprivation-2015.

[2] Charlson M, Pompei P, Ales K, MacKenzie C. A new method of classifying prognostic comorbidity in longitudinal studies: development and validation. J Chronic Dis. 1987;40(5):373–383.

[3] NHS Digital. Ambulatory Care Sensitive Conditions (ACSC); 2019. [accessed: 12.06.2020]. https://digital.nhs.uk/data-and-information/data-tools-and-services/data-services/innovative-uses-of-data/demand-on-healthcare/ambulatory-care-sensitive-conditions#top.

[4] Bankart M, Baker R, Rashid A, et al. Characteristics of general practices associated with emergency admission rates to hospital: a cross-sectional study. Emerg Med J. 2011;28(7):558–563.

[5] Walker A, Mason A, Quan T, et al. Mortality risks associated with emergency admissions during weekends and public holidays: an analysis of electronic health records. The Lancet. 2017;390(10089):62–72.

[6] Laudicella M, Li Donni P, Smith P. Hospital readmission rates: Signal of failure or success? Journal of Health Economics. 2013;32(5):909–21.

[7] Freemantle N, Ray D, McNulty D, et al. Increased mortality associated with weekend hospital admission: a case for expanded seven day services? BMJ. 2015;351(6):h4596.

[8] Aldridge C, Bion J, Boyal A, et al. Weekend specialist intensity and admission mortality in acute hospital trusts in England: a cross-sectional study. The Lancet. 2015;388(10040):178–186.

[9] Gilbert T, Neuburger J, Kraindler J, et al. Development and validation of a Hospital Frailty Risk Score focusing on older people in acute care settings using electronic hospital records: an observational study. The Lancet. 2018;391:1775–82.

[10] Royal National Orthopaedic Hospital and NHS England and NHS Improvement. Getting It Right FirstTime (GIRFT); 2016. [accessed: 9.12.2020]. https://www.gettingitrightfirsttime.co.uk/medical-specialties/geriatric-medicine/.
